# Deep brain stimulation for the treatment of Alzheimer's disease: A systematic review and meta-analysis

**DOI:** 10.3389/fnins.2023.1154180

**Published:** 2023-04-13

**Authors:** Alireza Majdi, Zhengdao Deng, Saeed Sadigh-Eteghad, Philippe De Vloo, Bart Nuttin, Myles Mc Laughlin

**Affiliations:** ^1^Exp ORL, Department of Neuroscience, Leuven Brain Institute, KU Leuven, Leuven, Belgium; ^2^Research Group Experimental Neurosurgery and Neuroanatomy, Leuven Brain Institute, KU Leuven, Leuven, Belgium; ^3^Neurosciences Research Center, Tabriz University of Medical Sciences, Tabriz, Iran; ^4^Department of Neurosurgery, University Hospitals Leuven, KU Leuven, Leuven, Belgium

**Keywords:** meta-analysis, systematic review, Alzheimer's disease, deep brain stimulation, cognition

## Abstract

**Background:**

One of the experimental neuromodulation techniques being researched for the treatment of Alzheimer's disease (AD) is deep brain stimulation (DBS). To evaluate the effectiveness of DBS in AD, we performed a systematic review and meta-analysis of the available evidence.

**Methods:**

From the inception through December 2021, the following databases were searched: Medline *via* PubMed, Scopus, Embase, Cochrane Library, and Web of Science. The search phrases used were “Alzheimer's disease,” “AD,” “deep brain stimulation,” and “DBS.” The information from the included articles was gathered using a standardized data-collecting form. In the included papers, the Cochrane Collaboration methodology was used to evaluate the risk of bias. A fixed-effects model was used to conduct the meta-analysis.

**Results:**

Only five distinct publications and 6 different comparisons (one study consisted of two phases) were included out of the initial 524 papers that were recruited. DBS had no impact on the cognitive ability in patients with AD [0.116 SMD, 95% confidence interval (CI), −0.236 to 0.469, *p* = 0.518]. The studies' overall heterogeneity was not significant (κ^2^ = 6.23, *T*^2^ = 0.053, df = 5, *I*^2^ = 19.76%, *p* = 0.284). According to subgroup analysis, the fornix-DBS did not improve cognitive function in patients with AD (0.145 SMD, 95%CI, −0.246 to 0.537, *p* = 0.467). Unfavorable neurological and non-neurological outcomes were also reported.

**Conclusion:**

The inconsistencies and heterogeneity of the included publications in various target and age groups of a small number of AD patients were brought to light by this meta-analysis. To determine if DBS is useful in the treatment of AD, further studies with larger sample sizes and randomized, double-blinded, sham-controlled designs are required.

## Introduction

Alzheimer's disease (AD) is a neurodegenerative disease responsible for ~60 to 80% of dementia cases and affecting at least 27 million people worldwide (Silva et al., 2019). Typical manifestations of amnestic AD, as opposed to non-amnestic AD, are progressive loss of episodic memory and cognitive function. This is followed by deficiency of language and visuospatial abilities accompanied by behavioral disorders including depression, apathy, and aggressiveness (Bateman et al., 2012).

Evidence suggests that structures located in the medial temporal lobe (MTL) such as the hippocampus, parahippocampal cortices, and amygdala undergo severe atrophy with AD progression (Ledig et al., 2018). MTL atrophy is linked to elevated amyloid-beta as well as tau and abnormal functional magnetic resonance imaging activity during memory encoding (Marks et al., 2017). These changes are shown to be linked to the severity of cognitive impairment and conversion from mild cognitive impairment (MCI) to AD (Yi et al., 2016). Besides, neuronal cell death and atrophy in the cholinergic nucleus basalis of Meynert (NBM) are associated with cognitive impairment in patients with AD (Liu et al., 2015). Further, it has been found that degradation of fornix may precede gray matter atrophy in AD and thus fornix measurements are a reliable indicator of conversion from preclinical disease to AD (Ringman et al., 2007).

Currently, AD remains incurable, and the search for new therapeutic agents has, to date, been tremendously unsatisfactory (Silva et al., 2019). This could be in part due to the fact that the pathogenesis of AD is still unclear. Apart from its neurodegenerative nature, AD can be viewed as a “neural circuit disorder” because of its impacts on several cortical and subcortical connections, particularly those involved in cognition and memory (Segtnan et al., 2019). In that light, modulation of the activity of neurons and related networks is of interest in AD (Laxton and Lozano, 2013). Deep brain stimulation (DBS) is one of the neuromodulation methods which are currently experimentally applied for the treatment of AD (Ringman et al., 2007; Segtnan et al., 2019). DBS modulates neuronal activity by delivering stimulation from an implantable pulse generator connected to electrodes implanted in a target area (Mirzadeh et al., 2016). Until now, three different locations have been tried as a target for the improvement of memory and cognition using DBS in AD. These are the NBM (Kuhn et al., 2015), the fornix (Laxton et al., 2010), and the ventral capsule/ventral striatum (VC/VS) region (Scharre et al., 2018).

Here, we perform a systematic review and meta-analysis of existing data to assess the outcome and of DBS in AD.

## Materials and methods

### Search strategy and selection criteria

The 2015 PRISMA guidelines were used to conduct this systematic review and meta-analysis (Shamseer et al., 2015). Two independent investigators systematically searched the Medline *via* PubMed, Scopus, Embase, Cochrane Library and Center for Reviews & Dissemination, and Web of Science databases using the terms “Alzheimer's disease,” “AD,” “deep brain stimulation,” and “DBS” and following search strategy: [TITLE-ABS-KEY (Alzheimer's AND disease) OR TITLE-ABS-KEY (AD) AND TITLE-ABS-KEY (deep AND brain AND stimulation) OR TITLE-ABS-KEY (DBS)] from inception until November 28, 2021. The references of included studies, a recent original article by Mao et al. (2018), and also a review by Aldehri et al. (2018) were screened for other potentially eligible cohorts. Disagreements were resolved by a third senior investigator. The search was limited to humans, original articles, and English studies. No restrictions were applied to the time of the study, age, gender, ethnicity, disease as well as follow-up length, disease severity, or subtype of AD. The inclusion criteria were (1) clinical trials or reports of DBS for patients with AD; (2) had a sample size of ≥2 included patients, and (3) original, published, and peer-reviewed articles. Based on these criteria, case reports were not included in this study.

### Data extraction and outcome measures

A standardized data collection form was used to extract the data from the included articles. Two independent authors extracted the data to avoid extraction errors. Discrepancies were resolved by discussion or a third senior author. Author names, year of publication, study design, sample size, age, gender, DBS location, type of cognitive tests used in the study, cognitive outcome(s), and neurological adverse events were extracted from the publications. Data in the table were expressed as numbers or mean ± standard deviation (SD). The most recent data with the longest follow-up duration were included in the case of multiple data (DBS treatment at different time points of (1, 3, 6, 12 months) emerging from the same population). The primary outcome measure of this study was the effect of DBS on cognitive function in patients with AD as assessed by the Mini-Mental State Exam (MMSE) and/or different subtests of the Alzheimer's Disease Assessment Scale (ADAS).

### Risk of bias assessment

The risk of bias (RoB) was assessed using the Cochrane Collaboration tool in the included studies. This tool is comprised of selection, performance, detection, attrition, and reporting bias items (Higgins et al., 2011). Various aspects of RoB such as random sequence generation, incomplete or selective reporting, allocation concealment, and blinding of participants and outcomes were assessed by two independent authors and any disagreement was resolved by discussion or third senior author.

### Statistical methods

The Comprehensive Meta-Analysis (CMA, version 2; Biostat, Englewood, NJ) and SPSS 26 (Armonk, NY: IBM Corp.) software packages were applied for data analysis. All data were expressed as mean ± SD. Meta-analysis was performed *via* the fixed-effects model, as the heterogeneity of the included studies was low (*I*^2^ < 25%) (Borenstein et al., 2010). The means of the conditions (pre vs. post) in each study were compared using the standardized mean difference (SMD). The *I*^2^ statistic was applied to assess the heterogeneity of the data and stratified to 25%, 50%, or 75%, groups as low, modest, and high, respectively. Publication bias was measured using funnel plots, trim and fill analyses, Begg and Mazumdar rank correlation, and Egger's regression intercept. However, it has been argued that funnel plots of the SMD plotted against the standard error (SE) are susceptible to misrepresentation and overestimation of the presence and degree of publication bias (Zwetsloot et al., 2017). Thus, these data were interpreted with caution.

If data on more than one outcome were reported by a citation we calculated a combined effect in outcomes while simultaneously avoiding bias (note that the precondition for this analysis is that the outcomes were reported from the same subjects) (Borenstein et al., 2009). The mean for various outcomes reported for a single cognitive domain was measured as,


(1) $$\begin{array}{l} \bar{Y}=\frac{1}{m}\left(\sum_{j}^{m}Y_{j}\right) \end{array}$$

where “*Y*” is the mean for effect sizes from different outcomes and “*m*” is the number of means. Nevertheless, the cumulative variance of these means was estimated as,


(2) $$V_{\bar{Y}}={\left(\frac{1}{m}\right)}^{2}var\left(\sum_{j=1}^{m}Y_{i}\right)={\left(\frac{1}{m}\right)}^{2}\left(\sum_{j=1}^{m}V_{i}+\sum_{j\neq k}\left(r_{jk\;}\sqrt{V_{j}}\sqrt{V_{k}}\right)\right)$$

where “*V*” is variance, and “*m*” is the number of variances in the formula. *p* < 0.05 was considered statistically significant in all analyses.

## Results

### Literature search

**Figure 1:**
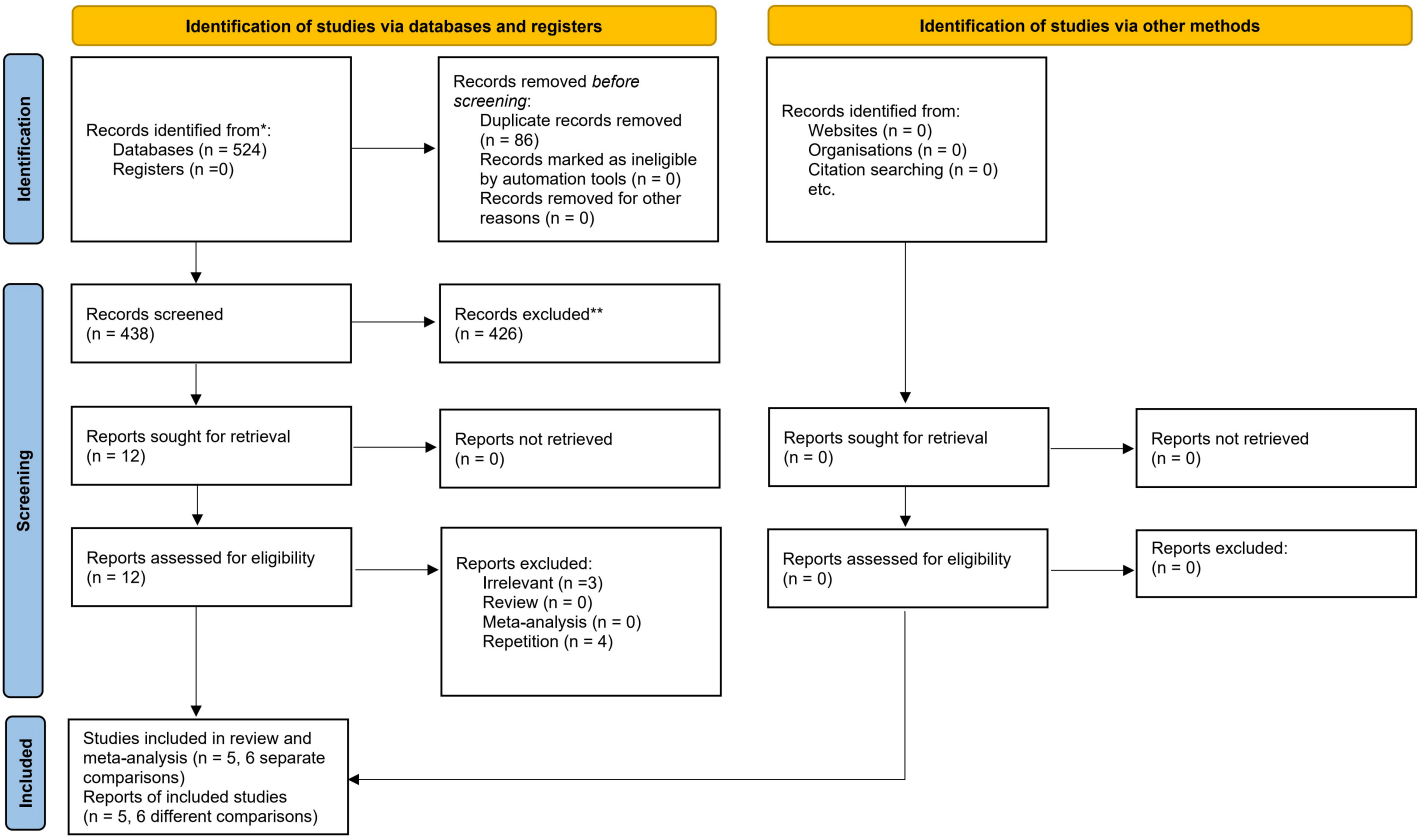
PRISMA flow diagram of the study. From: Page MJ, McKenzie JE, Bossuyt PM, Boutron I, Hoffmann TC, Mulrow CD, et al. The PRISMA 2020 statement: an updated guideline for reporting systematic reviews. BMJ 2021;372:n71. doi: 10.3389/10.1136/bmj.n71. For more information, visit: http://www.prisma-statement.org/.

A total of 524 publications were initially retrieved using the electronic search of the databases, of which 86 citations were excluded as they were duplicates. The remaining publications underwent title, abstract, and keywords screening resulting in the exclusion of additional 426 articles. Of the 12 publications that remained, three citations were excluded after the full-text screening. Another two publications (Laxton et al., 2010; Smith et al., 2012) were removed due to the assessment of the same patients as Sankar et al. (2015) study. Also, Kuhn et al. (2015) and Hardenacke et al. (2016) studies were not considered for inclusion due to using the same cohort of patients as Baldermann et al. (2018) study. Accordingly, five separate citations and six different comparisons were included in this systematic review and meta-analysis. The search strategy is shown in the flowchart of the study selection process (Figure 1).

### Study characteristics

The key descriptive characteristics of the eligible citations are briefed in Table 1. A total of 66 participants were included in this meta-analysis. Two studies had pre-post designs (Baldermann et al., 2018; Mao et al., 2018). One study was phase I (Leoutsakos et al., 2018) and one study was phase II (Leoutsakos et al., 2018) RCTs. One study was case-control (Sankar et al., 2015) and the other was a non-randomized phase I prospective open-label interventional trial (Scharre et al., 2018). The NBM was targeted in one study and the fornix (± hypothalamus) was stimulated as a target for DBS in three studies. One study chose the ventral capsule/ventral striatum (VC/VS) region as the DBS target (Scharre et al., 2018). The most common cognitive tests that were used to appraise the cognitive function of patients, were MMSE and ADAS-cog tests. Both neurological (stimulation-induced) and non-neurological- (surgical) adverse events were reported in the included publications (see discussion). The stimulation parameters in the included studies are presented in Table 2.

**Table 1:** Characteristics of the included studies in the systematic review and meta-analysis.

**References**	**Design**	**Number of patients**	**Male/female ratio**	**Age**	**MMSE of the patients**	**Disease severity**	**Medication (add-on-therapy)**	**DBS location**	**Cognitive assessment**	**Cognitive outcome**
Sankar et al. (2015)	Case-control	6	4/2	60.7 ± 6.1	22.3 ± 4.5	Mild to moderate	Cholinesterase inhibitors	Fornix	MMSE, ADAS-cog	Deterioration of cognitive function
Baldermann et al. (2018)	Pre-post design	10	4/6	66.9 ± 4.3	18.3 ± 3.8	Mild to moderate	–	NBM	MMSE, ADAS-cog, ADAS-mem	Improvement of cognitive outcomes in less-advanced disease
Leoutsakos et al. (2018)	Phase I and II RCT	42	–	Range: 45–85	–	Mild	Cholinesterase inhibitors	Fornix	ADAS-cog, CDRsb, CVLT-II, NPI	A possible cognitive benefit among older (>65) participants
Scharre et al. (2018)	Case-control	3	–	62.3 ± 11.8	22.67 ± 0.72	Mild to moderate	–	VC/VS	CDR	Less decline in CDRsb
Mao et al. (2018)	Pre-post design	5	2/3	59 ± 1.79	2.4 ± 1.15	Severe	Cholinesterase inhibitors, Chinese medication	Fornix	MMSE, MoCA, CDR	Improvement in some cognitive aspects

RCT, randomized clinical trial; NBM, nucleus basalis of Meynert; MMSE, mini-mental state examination; ADAS, Alzheimer's disease assessment scale; CDR, clinical dementia rating; DBS, deep brain stimulation; TMT, trail making test; MoCA, Montreal cognitive assessment; VC/VS, the ventral capsule/ventral striatum.

**Table 2:** The stimulation parameters in the included studies.

**References**	**Stimulation settings (mean** ±**SD)**		
	**Voltage (V)**	**Pulse width (μS)**	**Frequency (Hz)**
Baldermann et al. (2018)	2.62 ± 0.86	102.00 ± 25.29	15.50 ± 7.61
Leoutsakos et al. (2018)	NM	NM	NM
Mao et al. (2018)	1 to 5	90.00	130.00
Sankar et al. (2015)	3.0 to 3.5	90.00	130.00
Scharre et al. (2018)	NM	NM	NM

SD, standard deviation; NM, not mentioned.

### The effects of DBS on cognitive function in AD

**Figure 2:**
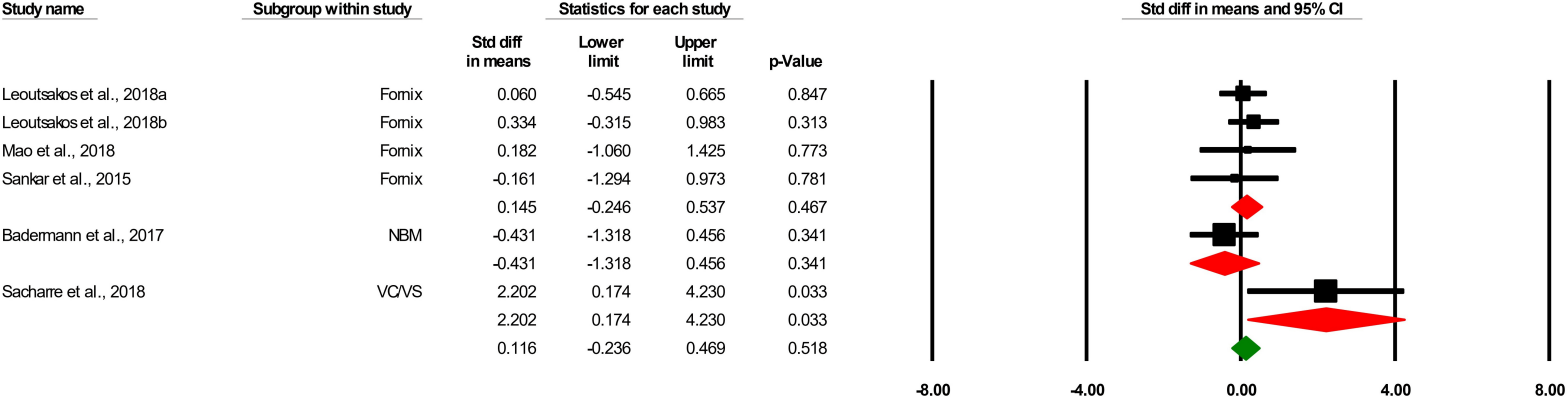
Forest plot of standardized mean difference (SMD) for the effect of deep brain stimulation on cognitive outcomes in different subgroups (target) in patients with Alzheimer's disease. The green square shows the overall pooled effect. Red squares show pooled effects in each subgroup. Black squares indicate the SMD in each study. Horizontal lines represent a 95% confidence interval (CI).

Six comparisons assessed the effects of DBS on cognitive function. Quantitative synthesis did not show any effects of DBS on cognitive function in patients with AD [0.116 SMD, 95% confidence interval (CI), −0.236 to 0.469, *p* = 0.518 (Figure 2)]. We found that the general heterogeneity of the studies was low (κ^2^ = 6.23, *T*^2^ = 0.053, df = 5, *I*^2^ = 19.76%, *p* = 0.284).

Three comparisons evaluated the effects of the fornix-DBS on cognitive function in AD. Meta-analysis revealed the non-significant effects of the fornix-DBS on cognitive function in AD patients (0.145 SMD, 95%CI, −0.246 to 0.537, *p* = 0.467; Figure 2). The heterogeneity of the comparisons was found to be low (not significant; I^2^ = 0.685, *T*^2^ = 0.000, df = 3, *I*^2^ = 0.00%, *p* = 0.877).

One comparison was on the effects of the NBM-DBS on cognitive function in AD and showed a non-significant effect of the intervention in this regard (−0.431 SMD, 95%CI, −1.318 to 0.465, *p* = 0.341).

### Adverse events

Neurological adverse events were altered mental status (two patients in one study), seizures or possible seizures (two patients in one study), agitation (one patient in one study) (Leoutsakos et al., 2018), mild pain at the implantable pulse generator site, headache at the incision site, transient visual neglect following surgery, and depression (frequency not reported) (Scharre et al., 2018). Non-neurological adverse events were also reported in fornix-DBS which were mainly autonomic and cardiovascular. These included sensation of warmth, sweating, flushing, increases in heart rate and blood pressure seen at stimulation >7 V. Sleep disturbances, changes in weight, metabolic or endocrine dysfunction, and/or hypothalamic abnormalities were not seen in any of the cases after 1 year of DBS. This was resolved by choosing chronic stimulation settings at 50% of the voltage threshold for side effects (Laxton et al., 2010). Another study reported malfunctioning plug-in connectors, demanding surgical revision of the corresponding implantable pulse generator. Inner restlessness at higher stimulation intensities (>5 V) was reported in one patient (Kuhn et al., 2015).

### Quality appraisal and publication bias

**Figure 3:**
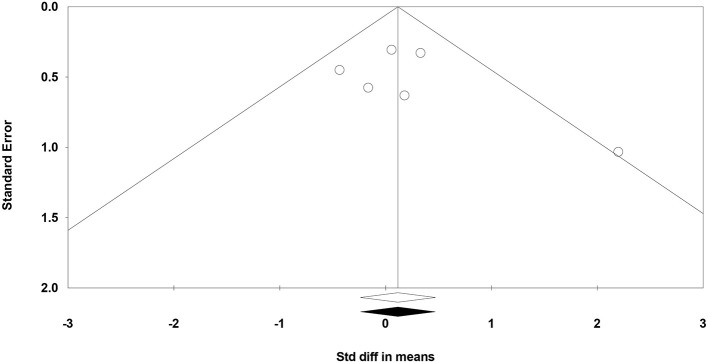
Funnel plot for the meta-analysis of studies of standard error by the standardized difference in mean cognitive scores.

The funnel plot (a bivariate scatter plot of SE against intervention effect) asymmetry raises the possibility of publication bias. In this study the funnel plot was reasonably symmetric, indicating a lack of publication bias (Figure 3). In line with that Begg and Mazumdar rank correlation did not reveal any evidence of publication bias (*p* = 0.452). Similarly, Egger's regression intercept did not show evidence of publication bias (*p* = 0.407). Trim and fill analysis showed no missing studies to the left or right of the mean.

**Figure 4:**
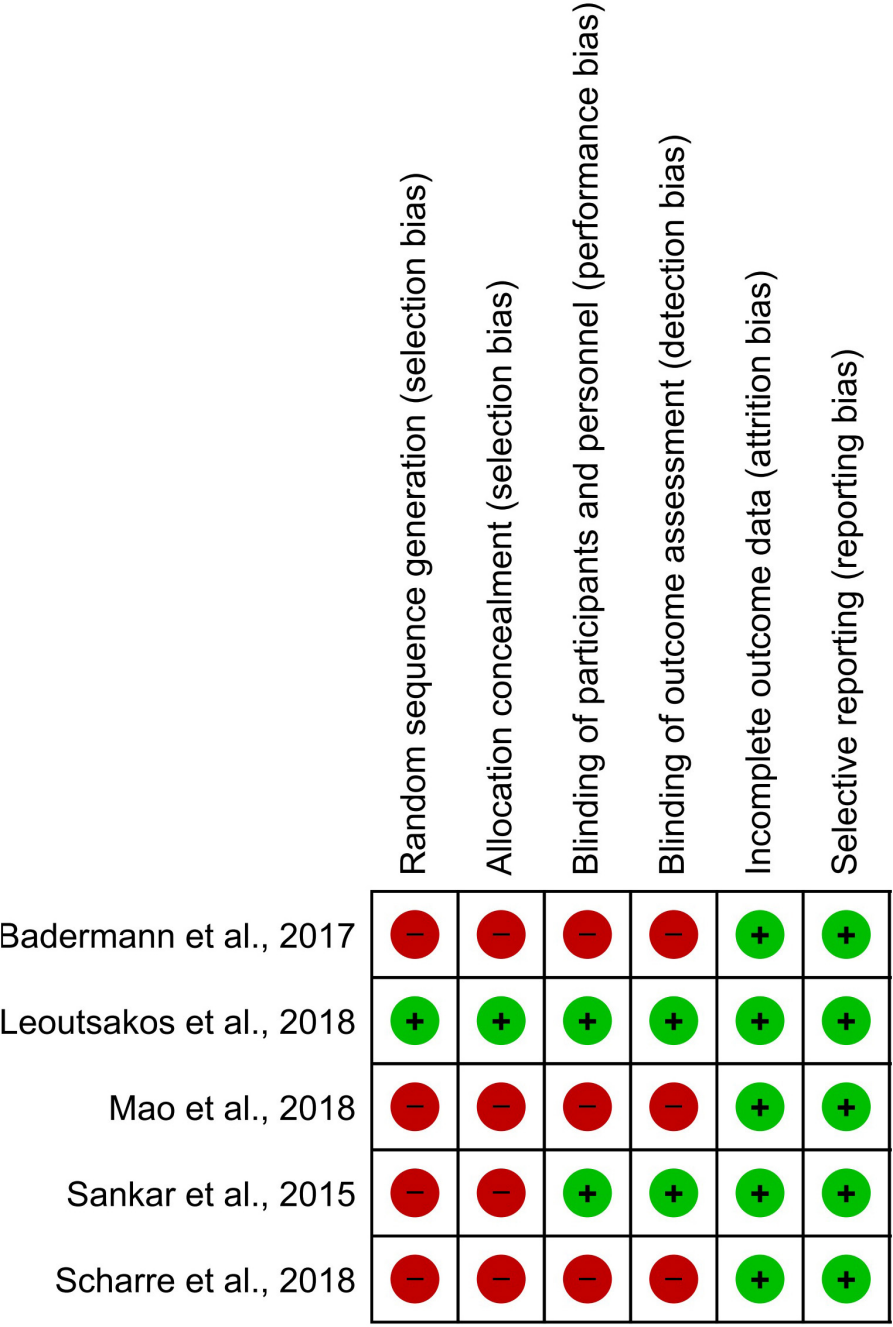
Different levels of risk of bias for each item in included studies. The Cochrane risk of bias tool was used for the detection of publication bias. In this color-coded ranking, the green color represents a low risk of bias and red high risk of bias.

However, the Cochrane Collaboration's tool showed that the majority of studies did not observe criteria for high-quality publications meaning that the participants were not randomized (*n* = 4), nor blinded (participants, researchers, and outcomes; *n* = 4). Allocation concealment was not also observed in these studies. Accordingly, selection, performance, and detection bias were apparent in these studies (Figure 4).

## Discussion

### General findings

This analysis showed that at the meta-level we were unable to detect a significant effect of DBS on the amelioration of AD-induced cognitive dysfunction. However, the stimulation targets and parameters (see Table 2) were very heterogeneous. The largest reported effect of DBS on AD was from one study which targeted VC/VS using low frequency stimulation. While three studies targeted the fornix and one studies targeted the NBM using different stimulation parameters (not always reported). Subgroup analysis showed that fornix-DBS (when analyzed separately) could not provide such an improvement. In general, the number of included studies and their quality (unblinded, non-randomized), as well as the number of participants, were low. The promising results from some studies, highlight the urgent need for the conduction of larger scale RCTs with bigger sample sizes and more standardized stimulation parameters to fully understand if DBS can become an innovative treatment for AD.

### A closer look at cognitive findings in individual studies

#### Fornix

Unfortunately, studies on the effects of the fornix-DBS in AD patients have yielded non-consistent results. Laxton et al., investigated the effects of 12-month fornix-DBS on six patients with mild AD. The results showed that fornix-DBS may ameliorate cognitive dysfunction and/or slow the rate of progression at 6 and 12 months in several patients. A robust correlation was found between cognitive function assessed by ADAS-cog and MMSE before surgery and the likelihood of response to DBS after the operation, with patients affected by milder forms of AD having a lower increase rate in ADAS-cog scores after 12 months of stimulation (Laxton et al., 2010). The Smith et al. study was the continuation of the previous study and revealed an increase in ADAS-cog scores ~2 points every 6 months-one year after the fornix-DBS in five patients with mild AD. However, cerebral glucose metabolism increased in two orthogonal networks of frontal-temporal-parietal-occipital hippocampal and frontal-temporal-parietal-striatal thalamic. The increase was in correlation with better global cognitive outcomes (Smith et al., 2012). Sankar et al. (2015) did analysis on the same cohort and showed that fornix-DBS in six patients with AD for 1 year resulted in an increase in ADAS-cog and a decrease in MMSE (meaning deterioration of cognitive function).

Similarly, in a 2-year follow-up of patients from the ADvance trial, fornix-DBS was employed to reduce the severity of cognitive dysfunction in patients with mild AD. However, this study was unable to show any differences in clinical outcomes in either phase of the study. Further analysis suggested a possible benefit from DBS treatment in participants older than 65 years (Leoutsakos et al., 2018). In another study, Mao et al. (2018) performed fornix-DBS in five patients with severe AD and showed partial improvement in performance in some cognitive tasks and aspects at an early stage of DBS.

#### NBM

The outcomes of studies on NBM-DBS have also been non-consistent. Kuhn et al., conducted a phase I RCT on six patients with AD and assessed the effects of the NBM-DBS in this group. The results showed worsening of ADAS-cog scores by an average of three points after 1 year of DBS, meaning a slow disease progression (worsening higher than three points are considered significant on this scale). The effects were claimed to be superior to those of anticholinergic medications. This study also showed that the MMSE score decreased only 0.3 points (almost stable) which was much lower a decrease than that of patients treated with pharmacotherapy (Kuhn et al., 2015). Baldermann et al., performed another analysis on the same cohort and showed that NBM-DBS for 1 year in 10 patients with AD stabilized MMSE score and improved (non-significant) ADAS-mem scores. However, ADAS-cog worsened in this sample after 1 year of DBS. This was in correlation with the fronto-parieto-temporal pattern of cortical thickness (Baldermann et al., 2018).

#### Ventral capsule/ventral striatum (VC/VS)

Only one study assessed the effects of VC/VS-DBS on cognitive outcomes in AD patients. Scharrre et al., conducted a non-randomized phase I prospective open label intervention of three patients for at least 18 months (27, 24, and 21 months) at the VC/VS target. All three subjects showed slower cognitive decline compared with the control patients in Clinical Dementia Rating scale Sum of Boxes (CDR-SB) score. DBS was tolerated in all subjects without any remarkable adverse events (Scharre et al., 2018).

### Rationale behind the currently-used targets

#### Fornix

Fornix is the predominant outflow tract and also a carrier of cholinergic axons from the septal area to the hippocampus. It has been found that fornix integrity predicts memory impairment and progression to AD (Mielke et al., 2012). Besides, lesions in the fornix cause memory impairment (Thomas et al., 2011). In that light, it is assumed that fornix-DBS may stabilize the Papez circuit activity and the default mode network (DMN), both of which show decreased metabolism during resting state in AD especially those with higher age and amyloid-beta burden (Greicius et al., 2004; Hardenacke et al., 2016). Fornix stimulation also causes a constant increase in the hippocampal volume as well as relative glucose metabolism, and a decrease in the mean rate of hippocampal atrophy in AD patients. Local volume expansion following fornix-DBS is not limited to the hippocampus and Papez circuit and is found in temporoparietal regions which are recognized to be atrophic in AD (Sankar et al., 2015).

#### NBM

It has been found that the basal forebrain cholinergic system, particularly that part residing in the NBM, undergoes severe atrophy as an integral part of AD-induced cognitive impairment (Leoutsakos et al., 2018). Also, Tau pathology is found in NBM with disease progression in AD (Tiernan et al., 2018). Thus, NBM seems to be a promising target in AD. NBM-DBS affects the cholinergic transmission, stabilizes activity in memory-associated circuits, induces neurotrophic factor production, and thus causes an improvement in cognitive function (Hardenacke et al., 2016).

#### VC/VS

Reminders can help AD patients with memory impairments, but caretakers have a hard time overcoming executive impairments such decreased curiosity, lack of initiative, apathy, distorted problem solving ability and poor self-regulation as well as decision-making. These functions are all executed by frontal networks including the entorhinal cortex, limbic structures (e.g., ventral striatum and nucleus accumbens) and frontal neocortex (Jack et al., 2010; Sachdev et al., 2013). By targeting VC/VS regions it is intended to alter frontal networks and influence executive functioning in AD subjects. Furthermore, as neurons in the VC/VS region undergo neurodegeneration after temporal regions in the course of AD, they may offer a superior target for modulation by DBS (Scharre et al., 2018).

### Stimulation-related parameters

Several stimulation parameters such as frequency, duration, start time, as well as location, unilateral/bilateral treatment, and current intensity should be considered when performing DBS in patients with AD; as these may affect the ultimate results in the study (Luo et al., 2021).

Other diseases have mainly been the basis for the selection of stimulation frequency in patients with AD. Until now, 20, 100, or 130 Hz frequencies have been used for DBS in AD patients. However, the optimal frequency remains unclear. In one animal study, the NBM-DBS of Aβ precursor protein/Presenilin1 (APP/PS1) mice led to better outcomes in higher frequencies (100 and 130 Hz) than lower frequencies (10 and 50 Hz) in the spatial memory assessment using the Morris water maze (MWM) (Huang et al., 2019). Others found that frequency did not influence the efficacy of DBS in AD; as 10 and 100 Hz led to the same results (Hescham et al., 2013).

### Disease stage and stimulation duration

The disease stage during which DBS is performed in patients with AD is also tightly associated with the treatment outcomes. An animal study performed on 4, 6, 9, and 12 month-old APP/PS1 mice using NBM-DBS showed that DBS at 4 months of age was linked to the best cognitive outcomes. However, DBS did not show such a robust effect at 9 and 12 months of age (Huang et al., 2019). A similar study in human beings revealed that NBM-DBS, performed in the early stages of AD or at younger participants, may be associated with decreased disease progression (Hardenacke et al., 2016).

Stimulation duration is another factor that may influence the results of DBS in AD patients. Evidence emerging from animal studies showed that both acute and chronic stimulation caused brain remodeling that persisted for a long time. Even 1 h of fornix-DBS improved cognitive outcomes and increased local volumes in mice. These changes lasted at least 45 days ruling out the hypothesis that the effects of DBS in AD are immediate (Gallino et al., 2019). However, the therapeutic outcomes of DBS were not found to be proportional to the treatment duration (Huang et al., 2019).

One animal study also showed that the effects of DBS in AD are influenced by current intensity. This study found that lower current intensities (50 μA), as opposed to higher ones (100 and 200 μA), are not associated with improvement of spatial memory in animals with AD (Hescham et al., 2013).

### Mechanistic view on DBS in AD

Several mechanisms may provide a rationale for the use of DBS in AD. First, low-frequency DBS on cholinergic neurons causes an increase in the release of acetylcholine from these neurons and improvement in cognitive function. Second, DBS resets the hippocampal θ rhythm, causes optimal encoding of input information, and improves memory in animals (Suthana et al., 2012). Third, the production and release of neurotrophic factors such as neural growth factor (NGF) may increase locally and in the ipsilateral neocortex by DBS in the target areas. NGF gene delivery to the basal forebrain has been shown to improve memory and decrease memory impairment in AD patients (Tuszynski et al., 2005; Hotta et al., 2009; Kuhn et al., 2015). Fourth, DBS is associated with the regulation of related neural networks in AD such as the Papez circuit and the default mode network that are typically impaired in these patients. An increase in the glucose metabolism of frontal-temporal-parietal-occipital and frontal-temporal-parietal-striatal-thalamic networks has been shown after fornix-DBS (Smith et al., 2012). Also, a fronto-parieto-temporal pattern of cortical thickness has been associated with NBM-DBS in AD patients (Baldermann et al., 2018). Fifth, DBS is linked to the reduction of Aβ and tau levels by regulation of glial cell activity (Vedam-Mai et al., 2016), decrease in tau phosphorylation as well as its oligomers accumulation in the CA1 region and a rise in tau autophagy-lysosomal degradation and the expression of synaptic proteins in 3xTg AD mice (Akwa et al., 2018). Sixth, DBS decreases the reactivity of astrocytes and microglia and neuronal loss in the cortex and hippocampus later after the electrode implantation and thus decreases neuroinflammation in the brain (Leplus et al., 2019). Other mechanisms such as modulation of changes in the gamma-aminobutyric acid (GABA) and glutamate systems, improvement of synaptic plasticity, promotion of neuron formation as well as regeneration, and regulation of brain-derived neurotrophic factor (BDNF) and vascular endothelial growth factor (VEGF) might also involve in the effects of DBS in AD (Luo et al., 2021).

### Limitations

This systematic review and meta-analysis had several shortcomings that may affect the results. First, the number of included studies in this meta-analysis was low. Second, the number of patients in each study and also in total was low. Third, the majority of studies had a pre-post design, were not randomized, nor blinded (low quality of included studies). It has been shown that studies with low quality tend to overestimate the results (Sadigh-Eteghad et al., 2017). Thus, there is an urgent need for the conduction of larger studies with bigger sample sizes and also randomized, double-blinded, sham-controlled design [see ADvance II Study (NCT03622905)]. Forth, stimulation parameters to perform DBS in AD are not unified. Parameters such as frequency, pulse width, stimulation target, start time, current intensity as well as duration, and unilateral/bilateral treatment are different between the included studies (Table 2). This might have caused the heterogeneity of the results.

## Conclusion

There is a growing interest in the therapeutic use of DBS in patients with AD suffering from cognitive decline. This meta-analysis highlighted the inconsistency and heterogeneity of the included publications. Other factors for concern were the low number of patients and lack of blinding and randomization in some studies. Any future studies investigating the use of DBS in AD patients should attempt to address these shortcomings.

## Data availability statement

The raw data supporting the conclusions of this article will be made available by the authors, without undue reservation.

## Author contributions

Study conception, design, and data collection: AM. Analysis and interpretation of results: AM and MM. Draft manuscript preparation: ZD, SS-E, BN, and PD. All authors reviewed the results and approved the final version of the manuscript.
